# Divergences between serum C-reactive protein and ferritin concentrations in canine pyometra

**DOI:** 10.1186/s12917-023-03630-3

**Published:** 2023-06-21

**Authors:** Jose J Ceron, Luis Pardo-Marin, Anna Wdowiak, Andrea Zoia, Marco Wochnik, Marek Szczubiał, Mariola Bochniarz, Fernando Tecles, Silvia Martinez-Subiela, Asta Tvarijonaviciute, Roman Dąbrowski

**Affiliations:** 1 Interdisciplinary Laboratory of Clinical Pathology (Interlab-UMU), Campus of Excellence Mare Nostrum, University of Murcia, Espinardo, Murcia, 30100 Spain; 2 Department and Clinic of Animal Reproduction, Faculty of Veterinary Medicine, University of Life Sciences, Głeboka 30st, Lublin, 20-612 Poland; 3 Division of Internal Medicine, San Marco Veterinary Clinic, Viale dell’Industria 3, Veggiano, Padua, 35030 Italy

**Keywords:** C-reactive protein, Ferritin, Divergence, Dogs, Pyometra

## Abstract

The main aim of this report was to investigate and compare the response of serum C-reactive protein (CRP) and ferritin, two positive acute phase proteins (APPs) which usually show an increase in inflammatory processes, in dogs with pyometra. For this purpose, two different studies were made. In the first one , both proteins were measured together in an APPs profile in 25 dogs with pyometra, 25 dogs with pancreatitis (as an example of a positive inflammatory control group), and in 25 healthy dogs. In the second study, to advance the knowledge of the changes and evolution of serum ferritin and CRP in dogs with pyometra after treatment, the concentrations of both APPs were analyzed in 30 dogs with pyometra at diagnosis and after ovariohysterectomy and in 10 clinically healthy female dogs before and after elective spaying. In both studies, bitches with pyometra showed significant increases in serum CRP, indicating an inflammatory condition, but not in serum ferritin despite being a moderate positive APP. This divergence between the dynamics of these APPs could be a useful tool for the suspicion of cases of canine pyometra.

## Introduction

Acute phase proteins (APPs) are proteins that change in their concentration during the onset of inflammation. The serum concentration of APPs can change depending on the intensity of the inflammatory response and the degree of stimulation of the immune cells system. The use of APP profiles (rather than individual tests) has been recommended in routine clinical practice [1]. Ideally, the profile should include at least one positive major, one positive moderate, and one negative APP. Major APPs show an early and high rise in concentration and a rapid decline, whereas moderate APPs require more time to increase and return to normal values. The main reason for recommending an APPs profile is due to the variable response pattern of these biomarkers occurring in some clinical situations which can provide useful information . For example, an increase in serum haptoglobin (Hp) concentration in dogs with C-reactive protein (CRP) within reference intervals can indicate the increased production of endogenous glucocorticoids, as it occurs in hyperadrenocorticism [2].

Pyometra is a common disease consisting of a bacterial infection and pus accumulation in the uterine lumen, affecting an average of 19% of all intact bitches [3]. In most cases, the disease occurs following oestrus and generally during the luteal phase [4, 5]. The bacterial uterine infection induces endometrial inflammation, and in some cases can lead to a systemic inflammatory response syndrome (SIRS) and the presence of bacteria in blood [6]. Based on this, evaluation of the inflammatory status is highly informative for the diagnosis and monitoring of pyometra.

At the Interdisciplinary Laboratory of Clinical Pathology of Murcia University (Interlab-UMU), a profile of APPs is included in the routine biochemistry panel for dogs, integrated by a major APP (i.e., CRP), two moderate APPs (i.e., Hp and ferritin), and two negative APPs (i.e., albumin and paraoxonase-1 [PON-1]). With the use of this profile, it has been observed that in cases of pyometra there is an evident increase in CRP and Hp as previously described [7]. In this disease, also increase in ferritin concentrations would be expected, as during inflammatory conditions, such as inflammation and infection, usually ferritin synthesis increases and serum iron (Fe) levels decrease making Fe less available for pathogens. This mechanism is the main protective strategy of the organism against infectious agents [8, 9]. However, using our laboratory’s APP profile, it has been observed that in canine pyometra, serum ferritin concentration usually remains within normal values and does not show the moderate increases expected in inflammation. This divergence response between ferritin and the other positive APPs, especially CRP, can potentially be used as an additional tool to raise the suspicion of pyometra in intact bitches when a biochemical profile of analytes including several APPs is interpreted.

The objective of this manuscript was to study and compare the CRP and ferritin response in canine pyometra to assess whether there is a consistent divergence in their response pattern in this disease. For this purpose, two observational studies were carried out. In the first one, the values of CRP and ferritin, together with the other three APPs that are include in our profiles (i.e., Hp, albumin and PON-1) in dogs with pyometra were compared with healthy dogs and with dogs suffering from another inflammatory condition such as pancreatitis. In the second study, the dynamics of CRP and ferritin in response to the surgical treatment of pyometra or elective ovariohysterectomy (OHE) of healthy bitches were evaluated.

## Materials and methods

In order to achieve the aims of this work, two studies have been performed.

### Study 1. APPs in pyometra at diagnosis compared to healthy dogs and dogs with pancreatitis

The database of 2 years (June 2020 to June 2022) of Interlab-UMU, University of Murcia, Spain, was analysed until obtaining 25 individuals for each of the three study groups of dogs, matched in sex (only females), age and body weight, with the following inclusion criteria, in addition to have the APP profile measured:

*Healthy dogs (control group).* This group included 25 entire bitches that were classified as healthy based on history, complete physical examination, haematology and biochemistry analyses.

*Dogs with pyometra (test group).* This group included 25 bitches diagnosed with pyometra. The diagnosis was based on the presence of compatible history or clinical signs (e.g., anorexia and/or pyrexia, polydipsia, polyuria, apathy, vaginal discharge and abnormal colour of mucous membranes), distended fluid-filled uterus on abdominal ultrasound and a surgical diagnosis of pyometra as previously described [7]. The diagnosis of pyometra was confirmed by postoperative histopathological examination of the uterus and ovaries.

*Dogs with pancreatitis (positive control group).* This group included 25 bitches diagnosed with acute pancreatitis. The diagnosis of pancreatitis was made on the basis of fulfilling all the following three criteria: (1) compatible clinical signs (such as anorexia, lethargy, abdominal pain, or vomiting), (2) increase serum 1,2-o-dilauryl-rac-glycero-3-glutaric acid-(6’-methylresorufin) ester (DGGR) lipase activity and (3) compatible abdominal ultrasonography with no other identifiable disease [10].

In all cases, and before any treatment was initiated, blood samples were collected from *vena cephalica* into tubes containing EDTA and tubes containing a clot activator (Vacutest Kima, Piove di Sacco, PD, Italy). The samples with EDTA were used for hematologic analysis performed with an hematology analyzer (Advia 120, Bayer, Barcelona, Spain) and verified by visualization of blood smears. Tubes with clot activator were centrifuged at 3000 *g* for 15 min, then the serum was transferred to Eppendorf tubes and used for biochemistry analysis, which included the APP profile. All samples were free of hemolysis and lipemia.

### Study 2. CRP and ferritin dynamics in response to elective ovariohysterectomy and surgical pyometra treatment

The study was conducted at the Department and Clinic of Animal Reproduction, in the Faculty of Veterinary Medicine of the University of Life Sciences in Lublin on a group of 40 bitches of different breeds, including mongrel dogs, with an age of 4–12 years and body weight of 12–50 kg, which were divided into two groups: the heathy client-owned control dogs (n = 10) admitted for elective OHE and the test group (n = 30) of dogs with pyometra. The diagnosis of pyometra was made as in the study 1, but in this case also uterine pus from all affected bitches was submitted for bacteriology. The study was performed by the regulations for animal protection (Animal Experimentation Act dated January 15, 2015) which agree with the European ethical principles for animal experiments.

The surgical procedures were carried out under general anesthesia. All dogs were pre-medicated with 40 µg/kg *im* medetomidine (Domitor^®^Pfizer, Warsaw, Poland). Anesthesia was induced with *iv* propofol (2 mg/kg, Plofed^®^ Polfa Warsaw, Poland) and maintained by inhalation of isofluorane in oxygen. For postoperative pain control immediately after surgery and 2 days postoperatively meloxicam (0.2 mg/kg, Metacam^®^ Boehringer Ingelheim, Warsaw, Poland) was administered by subcutaneous injections. During the first three postoperative days, bitches of both groups received intravenous infusions of 5% glucose, Ringer’s solution, and compound electrolyte solution in the amount of 10 ml/kg body weight. Cephalexin (Cefalexin 18%, ScanVet) was administered before surgery and for 5 days post-OHE at 10 mg/kg subcutaneously. Postoperative recovery was satisfactory in both groups of dogs. The skin sutures were removed in both groups of dogs on postoperative day 10 and the wound healed by the first intention in all dogs.

Before surgery (day zero, D0) and at day 3 (D3) and 10 (D10) after OHE, blood samples were collected from *vena cephalica* into silicone tubes with clot activator (Vacutest Kima, Piove di Sacco PD, Italy). The samples were centrifuged at 3000 *g* for 15 min and the supernatant was transferred to Eppendorf tubes and frozen at -80 °C until analysis. The samples were free of hemolysis and lipemia.

### Laboratory analyses

CRP was assayed using an immunoturbidimetric assay previously validated for dogs [11]. Haptoglobin was determined using a commercial colorimetric method (Tridelta Phase range haptoglobin kit, Tridelta Development Ltd) validated for dogs [12]. PON-1 was analysed based on an automated spectrophotometric assay using p-nitrophenyl acetate as substrate [13]. Albumin was measured by a commercially available spectrophotometric assay (Beckman Coulter, Beckman Coulter Life Sciences). Ferritin was assayed using a commercially available immunoturbidimetric test (Tina-quant Ferritin, Roche) designed for human samples. This assay was previously validated for dog showing intra and inter-assay imprecision below 15% [14].

### Statistical analysis

Results are shown as median (range) unless otherwise stated and were calculated using routine descriptive statistical procedures and software (Statistica 13 software, StatSoft, Poland; Graph Pad Prism, Version 6). The distribution of the studied variables was checked by using the D’Agostino & Pearson omnibus normality test. Given that majority of data did not follow Gaussian distribution, non-parametric tests were used. In study 1, the Kruskal-Wallis test followed by Dunns multiple comparison post-hoc correction was used to assess statistical differences among different groups. In study 2, the Friedman test followed by Dunns multiple comparison post-hoc correction were used to determine changes over time within the groups. Two-way ANOVA test followed by Sidak’s multiple comparisons post-hoc correction was used to assess the differences between the groups. The 5% error of inference and related significance level *P* < 0.05, indicating the existence of statistically significant dependencies or differences were accepted.

## Results

### Study 1

The 25 healthy female dogs had a median age of 8.5 years (range, 2–16 years) and a median body weight (BW) of 14.1 kg (range, 7.0–34.0 kg). Mongrel dogs were mostly represented (n = 12) in this group and the remaining dogs (n = 13) belonged to 9 different breeds. The 25 female dogs with pyometra had a median age of 8.2 years (range, 4−15 years) and median BW of 13.6 kg (range, 2.4–36.0 kg); these dogs were mostly mongrel (n = 12) and the remaining dogs (n = 13) belonged to 11 different breeds. All the dogs of this group had red blood cells indices inside the reference interval of our laboratory with the exception of 5 bitches that had mild normocytic normochromic non-regenerative anemia (with hematocrit values between 30 and 35% [reference interval, 37–58%]). These five dogs had reticulocyte hemoglobin content (CHr) and reticulocyte mean corpuscular volume (rMCV) inside the previously defined reference intervals, and therefore these values were not indicative of iron deficiency [15]. 

The 25 female dogs with pancreatitis had a median age of 10.1 years (range, 2–24 years) and median BW of 12.9 kg (range, 4.8–20.8 kg); 12 dogs were mongrel dogs and the remaining 13 dogs belonged to 10 different breeds. No statistically significant differences were detected between the three groups in terms of age and weight.

The median (range) of APPs serum concentrations of healthy dogs and dogs with pyometra and pancreatitis are reported in Table 1. Dogs with pyometra and pancreatitis showed statistically significantly higher serum CRP and Hp and lower serum PON-1 and albumin concentrations when compared with healthy dogs. Dogs with pancreatitis showed 3.1- and 3.5-fold higher serum ferritin concentrations than those observed in healthy dogs and dogs with pyometra, respectively (*P* < 0.001 in both cases), while no statistically significant difference was detected in serum ferritin concentrations between healthy dogs and dogs with pyometra (*P* > 0.05).

**Table 1:** Median (range) serum concentrations of acute phase proteins in healthy dogs and in dogs with pyometra and pancreatitis

Protein, unit (RI)	Healthy (n = 25)	Pyometra (n = 25)	Pancreatitis (n = 25)	P*
CRP, mg/L (1–12 mg/L)	5.3 (1.3–7.5)	118.0^a^ (54.0–354.6)	71.5^a^ (40.4–265.0)	< 0.001
Ferritin, µg/L (60–190 µg/L)	149.0 (81.0–187.0)	134.0^c^ (40.0–303.0)	465.5^a^ (136.0–3121.0)	< 0.001
Hp, g/L (1–3 g/L)	2.1 (1.4–2.8)	4.2^b^ (2.7– 5.7)	4.3^b^ (0.1– 4.9)	0.002
Albumin, g/dL (2.5–3.6 g/dL)	3.2 (2.6–3.5)	2.5^a^ (1.8–3.3)	2.2^a^ (1.3–3.0)	< 0.001
PON1, IU/L (3–4.3 IU/L)	4.0 (3.2–4.3)	2.6^a^ (1.2–4.4)	2.5^a^ (1.6–4.5)	< 0.001

*Kruskal-Wallis test

Letters indicate statistical significance between groups obtained with Dunn’s multiple comparisons test: ^a^, *P* < 0.001 vs. healthy; ^b^, *P* < 0.01 vs. healthy; ^c^, *P* < 0.001 vs. pancreatitis group

CRP, protein reactive C; Hp, haptoglobin; PON1, paraoxonase. RI, reference intervals. RIs of all APPs have been calculated using data of 50 healthy adult dogs

### Study 2

The control group comprised 10 healthy bitches with median age of 5.8 years (range, 4–11 years) and a median BW of 17.5 kg (range, 12.0–50.0 kg) undergoing OHE. Dogs were of five different breeds including mongrel. All were in diestrus as determined by history, clinical examination findings, and vaginal cytological examination findings. The test group comprised 30 bitches with a median age of 6.5 years (range, 4–12 years) and a median BW of 15.8 kg (range, 12–40.0 kg) of seven different breeds including mongrel dogs, admitted for surgical treatment of pyometra. *Escherichia coli* was isolated from the uterine pus culture in all cases. Postoperative recovery was satisfactory in both groups of dogs. The skin sutures were removed in both groups of dogs on postoperative day 10 and the wound healed by first intention in all 40 dogs. No statistically significant differences were detected between the two groups in terms of age and weight.

Median (range) data of APPs determined in the serum of healthy dogs and dogs with pyometra at D0, D3, and D10 after OHE are presented in Table 2. Serum CRP concentrations in dogs with pyometra were statistically significantly higher compared with healthy control dogs before surgery (D0) and at D3 (*P* < 0.001 and *P* < 0.01, respectively), while there was no difference between the two groups at D10. After surgery, a significant increase in serum CRP occurred at D3 and D10 compared to D0 in the healthy group (*P* < 0.05 for both comparisons), while at D10 serum CRP decreased compared to D0 in the dogs with pyometra (*P* < 0.01).

There were no significant differences in serum ferritin concentrations in dogs with pyometra compared to healthy dogs at D0 and D3. At D10, median ferritin concentrations significantly increased in both groups of dogs being statistically significantly higher than their initial values (*P* < 0.05 in healthy dogs; *P* < 0.01, in dogs with pyometra). In addition, on D10 after surgery, dogs with pyometra showed slightly higher circulating serum ferritin concentrations as compared with healthy dogs (*P* < 0.05).

**Table 2:** Median (range) serum CRP and ferritin concentrations in healthy dogs and in dogs with pyometra at D0, D3, and D10 post-ovariohysterectomy

Analyte	Group	D0	D3	D10	*P* ^*&*^
CRP, mg/L	Healthy	5.0 (3.05–10.8)	32.0 (22.7–40.1)^a^	14.6 (10.4–16.2)^a^	< 0.001
	Pyometra	99.5 (86.4-110.7)***	86.5 (76.4–95.0)**	34.6 (20.5–46.0)^b^	
Ferritin, µg/L	Healthy	137 (109.0-141.4)	146 (120.0-179.0)	191 (124.0-212.7)^a^	< 0.001
	Pyometra	145.7 (90.7-191.2)	151.0 (102.9–248.0)	214.7 (145.7-330.9)^b^*	

&, Two-way ANOVA. *, P < 0.05; **, P < 0.01; ***, P < 0.001 vs. same time between groups obtained with Sidak’s multiple comparisons test

^a^, P < 0.05 vs. D0 within the group; ^b^, P < 0.01 vs. D0 within the group obtained by Friedman test followed by Dunn’s multiple comparisons test

CRP, protein reactive C; D0, day before surgery; D3, day 3 post-ovariohysterectomy; D10, day 10 post-ovariohysterectomy

## Discussion

In this report, we described a divergence in the response of two serum-positive APPs, CRP and ferritin, in canine pyometra. This divergent response consists in the fact that serum CRP increases in pyometra above its upper reference interval, as previously reported [7], while serum ferritin, which should also be increased as it is a moderate APP, appears to be no different in comparison to healthy matched dogs, and in the majority of the cases was within the reference interval. Other authors previously reported divergences in the dynamics of two different APPs, as it appears in cases of hypercortisolism with an increase in Hp but a decrease in CRP [2] or in cases of intravascular haemolysis with a decrease in Hp but an increase in CRP [16]. A divergent pattern in APPs could therefore help to detect these conditions [17]. In the case of the pyometra, the presence of compatible history and clinical signs, an increase in serum CRP with serum ferritin within normal values could also be of help to suspect the presence of this disease. The divergent pattern of increased serum CRP concentrations with serum ferritin concentrations within reference interval was observed in the dogs with the pyometra in both studies that we performed, indicating consistency of this finding.

In study 1, three different APP patterns could be observed. (1) The pattern of healthy dogs, consisting of all APPs in the reference interval, indicating an absence of inflammation [1]. (2) The pattern of dogs with pancreatitis with an increase in all positive APPs and decrease in the negative APPs, as has been previously reported [10], due to the acute inflammation associated with this disease. (3) The pattern of dogs with pyometra with increases in all positive APPs except ferritin and a decrease in all negative APPs, that could help to suspect the presence of this disease.

Study 2 was performed to analyse the changes in serum CRP and ferritin in dogs with pyometra after surgical treatment. The magnitude of increase in CRP in dogs with pyometra compared with healthy dogs in this experiment and its dynamics of decrease after treatment was similar to a previous report in which the same procedure was made [7]. Serum ferritin concentrations in the dogs with pyometra were comparable to the healthy dogs, being for the majority of the cases within the reference interval, as in study 1. Interestingly at D10, when the pyometra was resolved, the serum ferritin concentrations showed an increase compared to D0, similarly to healthy dogs undergoing elective OHE, with average serum ferritin concentration well above the upper reference interval.

A possible reason for the divergence in the response between CRP and ferritin could be due to the different kinetics in their response. Alternatively, the findings of our report could also suggest that during the onset of pyometra some pathophysiological pathways would preclude the inflammatory increase of ferritin. In our experience, a similar pattern with major increases in serum CRP but serum ferritin within the reference interval has been observed in dogs with prostatitis or kidney infection by *E. coli* (data not shown). Since most of the pyometra are caused by *E. coli* [7], it could be postulated that the presence of *E. coli* could be involved in the lack of increase of ferritin found in pyometra. These suspicions would be in line with the previously reported observation that *E. coli* causes less alterations in iron metabolism than other bacteria [18]. However, further studies with a larger number of dogs and different *E. coli* infections should be made to corroborate this hypothesis. In the present study, only a few dogs with pyometra presented mild normocytic normochromic anaemia, a finding which is in line with previous reports [19], indicating that the inappropriately normal serum ferritin concentration found in this situation is not related to chronic blood loss. Nevertheless, these findings should be confirmed in additional studies with a larger population of dogs with different diseases and it would be also of interest to compare the diagnostic potential of the combination of serum CRP and ferritin concentrations with other tools used for the diagnosis of pyometra. In addition, the evaluation of the dynamics of these APPs in complicated cases of pyometra should also be explored.

This manuscript has three main limitations. First, ideally the subtype of ferritin, light (L) or heavy (H) that the assay used in this study quantifies should have been determined, knowing that H subunit is predominant in canine serum ferritin [20]. Hovewer, the increase of serum ferritin in dogs with pancreatits whould support the fact that the lack of serum ferritin increase in dogs with pyometra is not assay related. Second, the possible effect of different concentrations of estrogens and progesterone should have been explored in dogs with pyometra since they can influence endometrial histopathology and ultrastructural changes and possibly also APPs profile [21]. Third, in study 1, uterine pus from all affected bitches should have been submitted for bacteriologic analysis to support the hypothesis that *Escherichia coli* is involved in the lack of rise in serum ferritin concentration and also to evaluate how other bacteria types could influence the changes in the APPs. However, this was not possible given the study was retrospective. Therefore, further studies are needed to assess whether a different type of bacteria could influence concentrations of serum ferritin and other APPs. In general, it would be necessary to carry out a large-scale study that overcomes the aforementioned limitations to confirm the results of this study, which suggest that the use of ferritin and CRP could be useful in the diagnosis of canine pyometra.

In conclusion, dogs with pyometra present a divergence in the acute phase response with a major increase in serum CRP but not in serum ferritin that remains within reference intervals. This pattern, although should not be used as the only diagnostic tool, could be useful for the diagnosis of canine pyometra.

## Data Availability

The datasets generated and/or analyzed during the current study are not publicly available due to ethical and data protection reasons but are available from the corresponding author on reasonable request.
